# Community-Based Surveys for Determining the Prevalence of HIV, Chlamydia, and Gonorrhoea in Men Having Sex with Men in Hong Kong

**DOI:** 10.1155/2013/958967

**Published:** 2013-04-09

**Authors:** H. T. H. Wong, K. H. Wong, S. S. Lee, R. W. M. Leung, K. C. K. Lee

**Affiliations:** ^1^Stanley Ho Centre for Emerging Infectious Diseases, 2/F Postgraduate Education Centre, Prince of Wales Hospital, The Chinese University of Hong Kong, Shatin, Hong Kong; ^2^Special Preventive Programme, Department of Health, Centre for Health Protection, Hong Kong

## Abstract

*Background*. Community sampling of men having sex with men (MSM) for human immunodeficiency virus (HIV) and sexually transmitted infections prevalence studies poses challenges in view of problems in logistics and the hidden nature of MSM population. *Methods*. MSM in Hong Kong were recruited through social venues and the Internet. All participants were invited to complete a behavioural questionnaire and submit a urine specimen for HIV, Chlamydia, and gonorrhoea testing. *Results*. Totally, 994 MSM were recruited. No differences between venue and online-recruited respondents were identified regarding their demographics and infection status. The prevalence of HIV, Chlamydia, and gonorrhoea was 3.6% (95% CI: 2.6–5.0%), 4.7% (95% CI: 3.6–6.2%), and 0.2% (95% CI: 0.1–0.7%), respectively. Of all HIV cases, only 8.3% were aware of the infection; reflecting newly infected MSM were probably overrepresented. Some 58.3% had had HIV test within the past year, and 11.1% had CT/NG coinfection. HIV infection was associated with group sex [aOR: 2.67 (1.03–6.92)], receiving money for anal sex [aOR: 4.63 (1.12–19.18)], and unprotected anal sex with nonregular partners [aOR: 3.047 (1.16–8.01)]. *Conclusion*. Difference between venue- and online-recruited MSM was observed. A combination of sampling methods is complementary for epidemiology purpose. Overall, risk behaviours practised by undiagnosed HIV-positive MSM remains a cause for concern.

## 1. Introduction

Unprotected sexual contacts have remained one of the major routes of acquiring HIV and other sexually transmitted infections (STI) in the men who have sex with men (MSM) population globally [1–4]. In Hong Kong, despite the low HIV prevalence in the general population [5], a rising trend of new HIV reports in MSM has been observed over the last decade [3, 4]. Since 2006, HIV prevalence in MSM in the territory has been hovering around 4%, and there is concern for a continued increase with time [3, 4]. The research on risk factors is important to better inform future prevention strategies.

Behavioural factors aside, effects of bacterial STI on HIV transmission have been reported in various studies [6–8]. It has been suggested that the STI could enhance HIV's infectivity by disrupting hosts' epithelial surface and by recruitment and activation of HIV target cells during inflammatory response [7]. Of all STI, *Chlamydia trachomatis* (CT) and *Neisseria gonorrhoeae* (NG) are among the commonest pathogens that afflict MSM [2, 9, 10]. Infection caused by these organisms may increase HIV viral load in semen by up to 8-folds [10]. In Hong Kong, bacterial STI have remained the most prevalent diseases among male attendees of government STI clinics for years [11]. In 2011, 88% and 58% of all gonorrhea and non-gonococcal urethritis patients were male [11]. Nevertheless, interpretation of these data in context of sex between men is limited by the lack of paralleled behavioural data. 

While MSM who practice unprotected sex constitutes a population of potentially high risk of HIV/STI transmission, its characterization through targeted surveys is a tough challenge [12, 13]. Due to social stigmatization and cultural difference, MSM usually decide to remain hidden [13]. To date, most available MSM data are clinic based, reflecting a convenient yet biased sample [12]. Surveys of MSM through gay social venues or via the Internet are currently feasible [12, 14]. There are however limitations as regards specimen collection, storage, and transportation [15], while reliance on self-reported diagnoses collected online remains controversial [16, 17]. In order to determine the HIV/STI prevalence of Hong Kong MSM at the community level, we conducted a cross-sectional study, using both venue and Internet sampling, in 2011. Biological specimens were collected from all participants to measure the prevalence of HIV and urethral CT/NG infection.

## 2. Methods

Every 2 to 3 years, a cross-sectional HIV surveillance study on MSM has been conducted by the Department of Health of the Hong Kong Special Administrative Region Government with the collaboration of the academia and the community. In the year 2011, recruitment of MSM was made through two channels-gay social venues and the Internet. All participants were required to complete a behavioural questionnaire and submit a urine specimen for HIV/STI testing. Test results were released on a voluntary basis. Institutional approval for access to the data was sought from the Department of Health, in compliance with the Personal Data (Privacy) Ordinance. The conduction of the project was in accordance with the Declaration of Helsinki which indicated the interest of participants who took precedence over scientific interest. Participation of the study was entirely voluntary. For the venue survey, in order to minimize the disturbance in the adjacent venues and for simplicity's sake, verbal consent was obtained instead of a written consent. The trained interviewers explained the purpose and procedure of the study to the participants prior to each interview. Participants had the right to withdraw from the study at anytime. For the Internet survey, all Internet participants were required to read through a consent page before entering the core part of the study. All data and specimens contained no personal identifiers and were kept in strict confidence.

### 2.1. Sampling

Using time-location sampling [12], a representative sample of MSM was recruited from gay bars, saunas, and gay-popular beaches in Hong Kong. A presurvey mapping exercise was conducted to enumerate the target sample size of each venue by counting the client flows of each time-venue event (e.g., every Tuesday night 9–11 pm in bar A). MSM approached by trained peer workers were invited to complete an anonymous questionnaire using hand-held tablet computers. Respondents were required to submit a fresh first-catch urine specimen on site. An incentive of HKD$50 (USD$1 = HKD$7.8), in the form of a cash coupon, was offered to each successfully recruited MSM.

A study website (http://www.prismhk.org/) which had incorporated the identical questionnaire was launched immediately following the venue survey. It was publicized through online advertisements in gay websites and Internet forums and through internal circulars of gay community organizations. To avoid duplications, respondents were also asked if they had participated in the preceding venue survey. A unique study code was generated upon completion of the online questionnaire. With the code for matching, participants were asked to submit a urine specimen at a designated collection point. In order to attract MSM from heterogeneous backgrounds to participate in the Internet survey, all participants were entitled to receive an incentive of HKD$100 upon specimen submission. The collection points included outpatient clinics and community organizations located in every district in Hong Kong. 

### 2.2. Participants and Questionnaire

Eligible participants were male who (1) had been residing in Hong Kong during at least half of the time in the preceding 6 months or who were Hong Kong permanent residents (defined as holders of the Hong Kong Permanent Identify Card); and (2) had had anal or oral sex with another male in Hong Kong in the last 6 months. 

An anonymous bilingual (Chinese and English) questionnaire was constructed consisting of 9 parts, covering 4 main areas: (1) basic demographics, (2) access of prevention efforts (including contact with outreach service, receiving a condom, and history of HIV testing), (3) history of STIs and health seeking behaviors, and (4) pattern of risk behaviors (including frequency of sexual contacts, condom use rate, years of anal sex, number and types of sex partner, drug use and drug injecting history, and sex work engagement). The questionnaire was field-tested, amended, and finalized before administration at venues on tablet computer and online through the designated website. It took about 20 to 30 minutes to complete the whole questionnaire. 

### 2.3. Laboratory Testing

All urine specimens were refrigerated at 4°C after collection. HIV antibody testing was undertaken at the Public Health Laboratory Centre, Department of Health using an IgG antibody capture particle adherence test, followed by Western Blot confirmation. 

Two mL of each collected specimen was separated and transported to the Infectious Disease Laboratory of Stanley Ho Centre for Emerging Infectious Diseases, The Chinese University of Hong Kong for detection of *Chlamydia trachomatis (CT)* and *Neisseria gonorrhoea (NG)* infection by Gen-Probe APTIMA Combo 2 (AC2) assay. AC2 is a nucleic acid amplification test (NAAT) for the detection of both organisms [18]. 

### 2.4. Statistical Analysis

Data collected from the 2 sets of surveys were entered in computer, followed by collation and merging to form the final database. Only the data of participants who had submitted a urine sample were included in the analysis. Online respondents who also had enrolled in the venue survey were censored in the data analysis. Prevalence of HIV and the 2 STIs derived from the venue survey was adjusted by weighting the actual number of specimens received from each venue with the enumerated target sample size of each venue to avoid overestimation [19]. The profiles of the demographic, social, and behavioural characteristics were tabulated. Comparison was made between MSM tested positive with HIV and the rest and between venue-recruited respondents those recruited online. Between-group univariate associations were examined by using chi-square test and simple logistic regression. Significant variables by univariate analyses were then further evaluated by multivariate logistic regression models. All analyses were done by using SPSS 17.0 for Windows.

## 3. Results

### 3.1. Characteristics of Respondents

Between July and November 2011, a total of 996 respondents had completed the questionnaire and submitted a urine sample, 816 of which from surveys conducted at venues and 180 through the Internet. Overall, the respondents accounted for 27.2% of 2995 MSM approached at the venues. For the Internet arm, 379 completed questionnaires were returned, accounting for 11.2% of 3380 visiting the designated website (derived from the hit rate recorded during the study period). Two Internet-recruited respondents were excluded in data analysis because of duplications (Table 1). The venues where MSM were recruited were largely bars (*n* = 569), followed by saunas (*n* = 192) and beaches (*n* = 55). 

The median age of all participating MSM (*n* = 994) was 30 ± 9.4 years. Majority (90%) of the respondents were Chinese. Half (51.8%) had attained university education or above (Table 1). There was no statistically significant difference between the venue- and Internet-recruited respondents in terms of sociodemographic characteristics (Table 2). 

### 3.2. Prevalence of HIV and STI

Overall, 3.6% (95% CI: from 2.6% to 5.0%) and 4.7% (95% CI: from 3.6% to 6.2%), urine specimens were found HIV and CT positive, respectively. The adjusted HIV prevalence of the venue survey was 4.1% (95% CI: from 3.4% to 4.9%) and CT: 5.1% (95% CI: from 4.4% to 5.9%). For the Internet survey, both HIV and CT gave a prevalence of 3.3% (95% CI: from 1.6%–7.2%). NG was only found positive in 2 (0.2%, 95% CI: from 0.1% to 0.7%) specimens from the venue survey. No statistically significant difference was identified between the infection status of venue and Internet respondents (Table 2). Nevertheless, MSM-recruited online were more likely to have experienced STI symptoms in the past 6 months (60.9% versus 73.0%, *χ* ^2^ = 16.24, *P* < 0.001) (Table 2).

There were altogether 81 MSM who tested positive for HIV and/or either or both of CT and NG. Of these 80% were Chinese of age between 21 and 40. The HIV/STI-positive MSM had all attained at least secondary school education (Table 1). Chi-square tests did not reveal any significant association between HIV infection status and respondents' demographics (Table 3). Among the HIV-infected MSM, only 3 (8.3%) were aware of their HIV-positive status. Only 4 (11.1%) cases had CT/HIV coinfection, and none had concurrent infection of GC and other diseases (Table 1).

### 3.3. HIV/STI Testing Behaviours and Exposure to HIV Prevention Information

While some 80% of all respondents considered themselves as having “no to small risk” of acquiring HIV, over half had taken HIV test(s), and about 40% had their last test in the preceding year (Table 1). MSM recruited online and through venues gave a similar rate of HIV test in the preceding year (39.8% versus 41.6%, *χ* ^2^ = 0.19, *P* = 0.67). Online respondents were more likely to have their last HIV test (66.4% versus 38.2%, *χ* ^2^ = 30.21, *P* < 0.001) and to have received a free condom at a service run by a nongovernmental organization (NGO) (36.3% versus 7.6%, *χ* ^2^ = 40.81, *P* < 0.001). They were also more likely to have discussed HIV/STI/safer sex related topics with their gay friends during the last year (73.0% versus 60.9%, *χ* ^2^ = 9.29, *P* < 0.01). As regards the channels for receiving HIV prevention information, online participants were less likely to have been exposed to such messages through social venues (61.5% versus 37.5%, *χ* ^2^ = 27.62, *P* < 0.001) (Table 2).

### 3.4. Sexual Behaviours and Predictors for HIV Infections

While almost all participants (93.4%) had ever had anal sex with another man, venue respondents were more likely to report to have lost their analvirginity than their Internet counterparts (94.9% versus 86.5%, *χ* ^2^ = 16.38, *P* < 0.001) (Table 2). Comparing between MSM-recruited online and through venues, no statistically significant difference was identified in terms of the total number of sex partners and the engagement in group sex. Venue-recruited MSM were apparently more likely to have had unprotected anal intercourse (UAI) with both regular (51.6% versus 41.6%, *χ* ^2^ = 3.35, *P* = 0.07) and non-regular partner (79.5% versus 64.3%, *χ* ^2^ = 8.77, *P* < 0.01) and engaged in substance use during sex (36.0% versus 14.3%, *χ* ^2^ = 37.36, *P* < 0.001). Participants recruited from the Internet were more likely to have received money/other rewards for anal sex (5.2% versus 1.8X%, *χ* ^2^ = 5.41, *P* = 0.02) (Table 2).

In univariate analysis, concurrent CT/NG infection was associated with HIV infection, though this association did not reach statistical significance (OR: 2.54 (95% CI: 0.9–7.9); *P* = 0.08) (Table 3). Several behavioural factors remained significantly associated with HIV infection after controlling for age, CT/NG infection, and substance use and total number of sex partner in the past 6 months. These factors included having engaged in group sex (aOR: 2.67 (95% CI: 1.03–6.92); *P* = 0.04), having received money/other rewards for anal sex (aOR: 4.63 (95% CI: 1.12–19.18); *P* = 0.04), and having had UAI with non-regular sex partners in past 6 months (aOR: 3.047 (95% CI: 1.16–8.01); *P* = 0.02) (Table 3). 

## 4. Discussion

Our study was the latest and largest attempt to assess, on a community level, HIV and urethral CT/NG infections of MSM in Hong Kong. The results suggested an HIV prevalence of approximately 4%, a value that has remained unchanged compared to that reported in 2006-2007 (4.1%) [4]. One key question is how the prevalence of 4% could best be interpreted—it may mean a steady state resulting from virus transmission at the same or a reduced level due to different HIV health initiatives in the region over the years, or just a plateau indicating prevalent infections in the population with little new transmission. However, the figure did not correspond well with the observation of the tripling of new HIV reports in MSM in the territory over the past 10 years [4]. In addition to a rising HIV prevalence in neighboring Asian cities such as Chongqing (19.1%) [20], Taiwan (8.9%), and Jakarta (8.1%) [4]. From our results, only 3 of the 36 HIV-infected individuals captured in this study could correctly self-identify their own HIV status (Table 1). On the other hand, nearly 60% of all infected cases had their last HIV test performed within the preceding 1 year (Table 1). These observations suggested that a majority of the positive cases detected in this prevalence survey could be defined as newly infected. The 4% prevalence derived in the community surveys could be reflective of the incidence, rather than prevalence of HIV amongst MSM in Hong Kong. Instead of having a stable HIV prevalence, ongoing transmission of STI, including HIV, has probably been taking place, evidenced by the detection of asymptomatic bacterial STI (notably CT infection in 4.7% of the recruited MSM). Despite the vague association in our study between CT/NG infection and HIV positivity, the prevalence of CT/NG would still be supportive evidence for onward HIV transmission amongst Hong Kong MSM. 

The linkage between behaviours and HIV infection has been widely reported in the literature [2, 4, 12, 21]. In our study, despite the higher condom use rate for anal sex with non-regular partners, the odds ratio for HIV was still higher if there had been concurrent practice of unprotected anal sex with non-regular partners. In fact HIV transmission could occur with regular partners, against the background of the failure of the infected person to know their own status. Having engaged in group sex was also a significant risk factor associated with HIV infection, a finding which could be used to develop tailored intervention programmes. Our study also revealed that those who had received money or other rewards for anal sex (*n* = 19) were at higher risk of acquiring the disease. Although those who receive rewards for sex might not necessarily be working in the sex industry, this observation somehow echoed the notion that male sex workers (MSW) are a subpopulation of these vulnerable to HIV infection with unique health needs [22]. There is currently no published data on the HIV prevalence and risk profile of MSW in Hong Kong. Studies have shown that the HIV prevalence of MSW in neighboring Chinese cities varies from around 5% in Shenzhen to as high as 11% in Guangzhou [23, 24]. Since cross-border sex work is common in Hong Kong [25], the HIV epidemic of neighboring regions could have been bridged by MSW. Nevertheless, further exploration into the local culture of sexual transaction within the MSM community is needed to contextualize and justify our observation. 

Methodologically, our surveillance surveys had incorporated the use of two strategies (venue and Internet) for recruiting MSM in the community. Unlike overseas studies reporting differences between the two groups of MSM in terms of age, race, and education [26], no demographic difference was observed in our surveys (Table 2). This discrepancy might be due to the high penetration of Internet services in Hong Kong which may have minimized the digital divide among MSM in the city. There were, in fact, some subtle differences between MSM separately recruited. Apparently online-recruited MSM showed a lower consistency in condom use with both regular partners and non-regular partners. They were also more likely to have engaged in sex that involved money or rewards. Also, a higher percentage of them experienced STI symptoms in the past 6 months than their venue counterparts. These findings suggested that MSM recruited from the Internet were more likely to be practising certain risk behaviours, or they were simply more willing to “tell the truth” as social desirability bias was not a concern when interviewers were not present [27]. The latter also reminded us to be on guard against misinterpreting results from the venue survey-if risk behaviours could be socially biased, then so could the health seeking behaviours such as the experience of HIV testing. Besides, one interesting observation that should not be missed was the differences in gay community attachment. Online MSM were far less likely to receive HIV prevention messages in gay venues but probably more receptive of initiatives of NGO (Table 2). As NGOs have assisted in the promotion of the online survey, cautions must be exercised when evaluating behavioural profiles.

There were a few limitations in the conduct of the surveys and their analyses. The predominance of incident cases in the HIV-positive subjects is a cause for concern. The unsuccessful recruitment of participants with known HIV diagnosis may be explained by one or more of the three reasons: Firstly, some MSM might have treated our study as an alternative way of HIV testing and thus been discouraged from the participation. Secondly, HIV-positive MSM might have changed their networking pattern and were therefore not identifiable through conventional channels. Finally, diagnosed individuals may be afraid to disclose their infection status due to stigma towards HIV/AIDS, which have been reported elsewhere and in Hong Kong [28, 29]. A similar community survey conducted in Pittsburgh reported that community-reached MSM who refused to take part in the study were significantly more likely to be HIV positive [30]. People who had experienced genitourinary symptoms were also more likely to be reluctant to participate [30]. This could also explain the significantly fewer number of venue-recruited participants with STI symptoms compared to the Internet arm in our study (Table 2). On the other hand, interpretation of the prevalence of STI (4.7% for CT and 0.2% for NG) could be a challenge when previous data were not available. In clinic-based studies elsewhere, the prevalence ranged from around 2% to 15% for CT and 1% to 17% for NG [9, 10, 31, 32]. NG infection was far less common than CT infection among community-recruited MSM [30, 33, 34]. This may be because of the symptoms frequently accompanying urethral NG infection, which might have urged infected individuals to seek medical treatments before being captured in surveys [35]. 

From the liaisons with venue owners during the preparation phase of the survey, it appeared that most owners were preoccupied with concerns about whether the survey would disturb their business. As the primary goal of our study was to snapshot the disease prevalence and HIV risk behavioural patterns among the MSM community, a compromise was made regarding the design of the questionnaire. Certain behaviours were not measured in details for the sake of a shorter administration time. Behaviours such as group sex and anal sex that involved the transaction of money/other rewards were dichotomized into “ever/never practiced” in the study and were seen as factors that predict HIV infection. However, simply to view these sexual behaviours as “risk factors” may mask the true reason that constitutes to the epidemic, when unprotected sex instead of the sex itself is the fundamental cause of disease transmission. Other than assessing the usage of condom, future studies may also address the underlying cultural, social, and structural forces acting on the decision of condom use. Typing partners into fixed categories—regular/non-regular—might also be problematic because sexual relationships might not be a binary phenomenon. These all suggest that the complement of qualitative work would have better informed, enhanced and extended the interpretations of our quantitative data. Last but not least, all behavioural data were self-reported by the respondents and were subjected to recall biases. The suboptimal response rate of the study (especially of the Internet survey) might have also impacted the generalizability of the results. Despite all these concerns, our study still met its objective of providing an updated overview of the HIV situation in MSM in Hong Kong. 

Our study also shed light on many possible directions for future investigation and other prevention opportunities. In order that HIV and risk behaviours can be tracked longitudinally and consistently, the mechanism for surveillance would need to be maintained, improved and regularly evaluated. This would require the continued support and cooperation between individuals, community organizations, and stake holders in the community (e.g., owners of social venues). Conducting such activities on ad hoc basis is clearly counterproductive. Apart from these, exploring and piloting innovative means of MSM surveys should be part of an ongoing effort in improving epidemiologic surveillance. The Internet arm of our survey should be treated as the beginning of a new mechanism, rather than a completed task on its own. Some subpopulations, and the culture and power dynamic within them, are currently underrepresented in surveillance activities. MSM who practice anal sex for money/other form of reward is a case in point. A systematic evaluation of the characteristics of MSM recruited from different channels (e.g., venues other than bars and saunas) may help reveal hidden network of MSM with different risk profile of public health importance. Qualitative enquiry can also expand the scope of the surveillance by contextualizing the quantitative data.

## 5. Conclusion

To conclude, our study managed to estimate the prevalence of HIV and selected STI prevalence of MSM in Hong Kong by the adoption of two different sampling methodologies. Compared to venue-based sampling, Internet appears to target MSM with a different set of social and behavioural characteristics, despite common demographic backgrounds. HIV infection was associated with selected risk behaviours and coinfection of CT/NG was not common. The HIV prevalence derived could have underestimated the true situation and should be interpreted in conjunction with epidemiologic and qualitative data collected through other channels. 

## Figures and Tables

**Table 1:** Characteristics of all respondents.

	All respondents (*n* = 994) *n* (%)	Infected individuals	
		HIV (*n* = 36) *n* (%)	CT/NG (*n* = 49) *n* (%)
*Demographics *			
Age			
≤20	95 (9.6)	2 (5.6)	3 (6.1)
21–30	417 (42.0)	17 (47.2)	21 (42.9)
31–40	327 (32.9)	13 (36.1)	20 (40.8)
≥41	155 (15.6)	4 (11.2)	5 (10.2)
Median age (±SD)	30 (±9.4)	30 (±10.2)	31 (±7.0)
Mode of recruitment			
Venue	816 (82.1)	30 (83.3)	43 (87.8)∗
Internet	178 (17.9)	6 (16.7)	6 (12.2)
Ethnicity			
Chinese	895 (90.0)	32 (88.9)	45 (91.8)
Non-Chinese Asian	36 (3.6)	2 (5.6)	2 (4.1)
White	44 (4.4)	2 (5.6)	1 (2.0)
Others	19 (1.9)	—	1 (2.0)
Highest Education attained			
Primary or below	7 (0.7)	—	—
Secondary/post-secondary education^†^	468 (47.1)	24 (66.7)	24 (49.0)
University or above	515 (51.8)	12 (33.3)	25 (51.0)
Others	4 (0.4)	—	—
*Time of last HIV test *			
≤3 months	144 (14.5)	5 (13.9)	9 (18.4)
3–12 months	255 (25.7)	16 (44.4)	21 (42.9)
>12 months	245 (24.6)	5 (13.9)	9 (18.4)
Never/Refused to answer	350 (35.2)	10 (27.8)	10 (20.4)
*Attitude on HIV *			
Self report HIV status			
Positive	5 (0.5)	3 (8.3)	—
Negative	520 (52.3)	17 (47.2)	29 (59.2)
Refused to answer	469 (47.2)	16 (44.4)	20 (40.8)
Perceived HIV risk			
No to small risk	833 (83.8)	21 (58.3)	37 (75.5)
Some to high risk	130 (13.1)	8 (22.2)	8 (16.3)
Missing	31 (3.1)	7 (19.4)	4 (8.2)
*Concurrent diseases infection *			
CT	—	4 (11.1)	—
NG	—	0	—
*Overall diseases prevalence (%) [95% CI]* ^ #^			
HIV	3.6 [2.6–5.0]	—	—
CT	4.7 [3.6–6.2]	—	—
NG	0.2 [0.1–0.7]	—	—

^*^Includes 41 CT infections and 2 NG infection.

^†^Post-secondary education includes associate degree, diploma, or other certificate courses.

^#^After adjusting by weighting the actual number of specimens received from each venue with the enumerated target sample size of each venue, disease prevalence for venue survey: HIV: 4.1% (3.4–4.9); CT: 5.1% (4.4–5.9); NG: 0.2% (0.1–0.9).

**Table 2:** Comparing participants recruited from venues and the internet.

	Venue (*n* = 816) *n* (%)	Internet (*n* = 178) *n* (%)	*χ* ^2^	*P* value
Demographics				
Age ≤ 30	419 (51.3)	93 (52.2)	0.47	0.83
Being Chinese	731 (89.6)	164 (92.1)	1.06	0.30
Having secondary education or below	225 (27.6)	60 (34.1)	2.94	0.09
Diseases pattern				
HIV positive	30 (3.7)	6 (3.4)	0.39	0.84
CT/NG positive	43 (5.3)	6 (3.4)	1.12	0.29
Experienced any STI symptoms in past 6 months^#^	45 (5.5)	25 (14.0)	16.24	**<0.001** ∗
HIV testing behaviours				
Had an HIV test in past 1 year	325 (39.8)	74 (41.6)	0.19	0.67
Last HIV test at a NGO^b^	208 (38.2)	75 (66.4)	30.21	**<0.001** ∗
Exposure to HIV prevention interventions in past 1 year				
Received HIV prevention messages in gay venues	390 (61.5)	54 (37.5)	27.62	**<0.001** ∗
Received a free condom at a NGO	40 (7.6)	37 (36.3)	40.81	**<0.001** ∗
Discussed HIV/STI/safer sex with gay friends	496 (60.9)	130 (73.0)	9.29	**<0.01** ∗
Risk behaviours				
Ever had anal sex	774 (94.9)	154 (86.5)	16.38	**<0.001** ∗
Had >5 anal sex partner in past 6 months^d^	182 (28.0)	45 (33.6)	0.68	0.41
UAI with regular partner in past 6 months^d^	220 (48.4)	59 (58.4)	3.35	0.07
UAI with nonregular partner in past 6 months^e^	75 (20.5)	30 (35.7)	8.77	**<0.01** ∗
Used alcohol/illicit drugs during sex in past 6 months^c^	234 (36.0)	19 (14.3)	37.36	**<0.001** ∗
Received money/rewards for anal sex in past 6 months^c^	12 (1.8)	7 (5.2)	5.41	**0.02** ∗
Engaged in group sex in past 6 months^c^	148 (22.7)	23 (17.2)	2.00	0.16

^a^If ever received a condom in past 1 year; ^b^If ever tested;^ c^If ever had sex in past 6 months; ^d,e^UAI: unprotected anal intercourse; If ever had a regular/non-regular partner in past 6 months.

^*∧*^Others including government clinics, private clinics, self test at home, and other unspecified venues.

^#^STI symptoms include abnormal urethral discharge, dysuria/discomfort during urination, blister/abnormal growth/ulceration and other discomfort overgenital/anal area.

∗ *P* value < 0.05.

**Table 3:** Risk factors for HIV-positive status.

	OR^a^	*χ* ^2^	*P* value	aOR^††b^	*P* value
Demographics					
Age ≤ 30	1.05 (0.54–2.05)	0.02	0.88	—	
Chinese ethnicity	0.88 (0.31–2.5)	0.06	0.81	—	
Having secondary education or below	1.25 (0.62–2.53)	0.38	0.54		
Method of recruitment					
Venue	1.094 (0.45–2.67)	0.04	0.84	—	
STI pattern					
Concurrent CT/NG infection	2.54 (0.90–7.90)	3.05	0.08	—	
Experienced STI symptoms in past 6 months	1.21 (0.36–4.05)	0.10	0.76	—	
Having diagnosed with STI in past 6 months	0.85 (0.11–6.44)	0.02	0.88	—	
Sexual behaviours in past 6 months					
Having >5 anal sex partners	**2.21 (1.1**–**4.4)**	5.39	**0.02** ∗		
Having engaged in group sex	1.9 (0.93–4.00)	3.24	0.07	**2.67 (1.03**–**6.92)**∗	**0.04** ∗
Having used alcohol/illicit drugs when having oral/anal sex	1.13 (0.54–2.34)	0.10	0.75		
Received money/other rewards for anal sex	**6.80 (2.12**–**21.76)**	13.78	**<0.001** ∗	**4.63 (1.12**–**19.18)**∗	**0.04** ∗
Had UAI with regular partner^c^	1.78 (0.73–4.31)	1.66	0.20		
Had UAI with nonregular partner^c^	**3.52 (1.42**–**8.70)**	8.28	**<0.01** ∗	**3.047(1.159**–**8.011)**	**0.02** ∗

^a^OR: univariate odds ratio; ^b^aOR: adjusted odds ratio; ^c^UAI: unprotected anal intercourse.

∗ *P* value < 0.05.

^††^Adjusted for age, concurrent CT/NG infection, and substance use and total number of sex partner in the past 6 months.
